# Antibacterial and antifungal activities of the endemic species *Glaucium vitellinum* Boiss. and Buhse

**Published:** 2015

**Authors:** Mina Mehrara, Mehri Halakoo, Mojdeh Hakemi-Vala, Seyyde Jamal Hashemi, Jinous Asgarpanah

**Affiliations:** 1 *Department of Pharmacognosy, Faculty of Pharmacy, Pharmaceutical Sciences Branch, Islamic Azad University, Tehran – Iran (IAUPS)*; 2 *Microbiology Department, Medical School, Shahid Beheshti University of Medical Sciences, Tehran, Iran.*; 3 *Department of Medical Mycology and Parasitology, School of Public Health, Tehran University of Medical Sciences, Tehran, Iran.*

**Keywords:** *Glauciumvitellinum*, *Papaveraceae*, *Antibacterial*, *Antifungal*

## Abstract

**Objectives:** Belonging to Papaveraceae family, *Glaucium vitellinum* is one of the Persian endemic plants which has not been investigated biologically. The present paper focused on the assessment of the antibacterial and antifungal activities of the total methanol extract and alkaloid sub-fraction of the flowering aerial parts of *G. vitellinum*.

**Materials and Methods:** The antibacterial and antifungal activities were investigated using cup plate method and disc diffusion assay, respectively. The MIC values of the active samples were determined using micro plate dilution method.

**Results:** The crude extract and alkaloid sub-fraction of *G. vitellinum* had significant inhibition activity on the growth of *S. aureus* and *S. typhi*. From antifungal assay, it is concluded that only the yeast *C. albicans*, showed a high sensitivity to the extract and especially to the related alkaloid sub-fraction.

**Conclusions:** Regarding the results, *G. vitellinum* could be employed as a natural antibacterial and antifungal agent against *S. aureus*, *S. typhi*, and *C. albicans,* respectively. Moreover, based on the results of this study, further in vivo and ex vivo confirmatory tests for total methanol extract and alkaloid sub-fraction are recommended.

## Introduction

The genus *Glaucium* (Papaveraceae) comprises about 25 species of annual, biennial or perennial herbaceous flowering plants in the world of which 11 species are found in different parts of Iran, particularly, is one of this genus origin centers with several endemic and native species, locally called ‘Shaghayegh’ (Mozaffarian, 2006 ▶). 

Several *Glauciem *species are used in folk medicine as hypnotic, laxative, anti-inflammatory, antihyperglycemic (Morteza-Semnani et al., 2002 ▶) and antispasmodic (Al-Khalil et al., 1991 ▶) agents as well as for antiseptic and astringent properties as a topical remedy especially in dermatitis (Morteza-Semnani et al., 2004 ▶). It has also been used for relieving warts (Bournine et al., 2013 ▶). The diversity, species richness and variation as well as chemical properties have recently led to much research on the genus *Glaucium*. Phytochemical investigations have reported isoquinoline alkaloids including aporphines, protopines, protoberberines and proaporphines as the major constituents of *Glaucium *species (Ivanovska et al., 1996 ▶). 

*G. vitellinum* is one of the endemic perennial species distributed in central parts of Iran in rocky mountains. It is an almost glabrous perennial herb with large yellow flowers and elongated capsule. Each of four yellow petals has little spots on the base. Blooms appear from April to July (Ghahreman, 1985 ▶). Literature survey revealed that *G. vitellinum* has just been phytochemically investigated and isocorydine, protopine, dicentrine, tetrahydropalmatine, muramine, bulbocapnine and glaucine have been identified in this plant (Shafiee et al., 1977 ▶). As other *Glaucium* species have shown significant antibacterial and antifungal activities (Morteza-Semnani et al., 2005 ▶; Coşar et al., 1981 ▶; Morteza-Semnani et al., 2003 ▶), we prompted to evaluate the antibacterial and antifungal activities of total methanol extract and alkaloid sub-fraction of *G. vitellinum* flowering aerial parts for the first time and investigate the biological basis for its folkloric application as an antiseptic and antifungal agent.

## Materials and Methods

**Plant material**

Fresh flowering aerial parts of *G. vitellinum* were collected in June 2013 from the mountain areas of Khansar County, Isfahan Province, Iran: (33° 15′ N 50° 20′ E, 2600 m). Specimen was identified by Dr. Gh. Amin and voucher was deposited in the herbarium of Pharmaceutical Sciences Branch, Islamic Azad University (IAU), Tehran under code number 1608 AUPF.

**Extraction Procedure**

Two kg of the air-dried grounded plant was extracted by percolator apparatus using methanol. The extraction was repeated for three times. The extract was concentrated by rotary evaporator apparatus and the solvent was removed to produce a dark green gummy solid. An adequate part of the resulting extract was kept in a sterile vial in a dark and cool place for further tests. The remains were extracted for total alkaloids. Two hundred ml of acetic acid-water (50:50) was added to the residue and the mixture was filtered. The filtrate was extracted with petroleum ether (5×100 ml) to remove colored materials. The aqueous layer was then made alkaline with 25% ammonia and extracted with chloroform (5×150 ml). Evaporation of the solvent gave a crude mixture of alkaloids (alkaloid sub-fraction). 

**Test organisms**

One Gram-positive bacterium *Staphylococcus aureus* (PTCC1431) and four Gram-negative bacteria including *Klebsiella pneumonia* (PTCC1053), *Escherichia coli* (PTCC25922), *Salmonella typhi* (PTCC1609) and *Pseudomonas aeruginosa* (PTCC25823) were obtained from Persian type culture collection (PTCC) of Iranian Research Organization for Science and Technology.

Fungal strains including *Trichophyton mentagrophytes, T. rubrum, Aspergillus flavus, Epidermophyton flucossum, Microsporum canis, *and the yeast *Candida albicans* were the nine different clinical isolates of pathogenic fungi and yeast taken for this study.

**Antibacterial activity**

Antibacterial activities of the methanol crud extract and the alkaloid sub-fraction of *G. vitellinum* were investigated against five bacterial strains by the cup plate method. An overnight bacterial culture equal to 0.5 McFarland standard (1.5 × 10^8^ CFU/ml) was used to culture on Muller-Hinton agar plates (Fazly-Bazzaz et al., 2005 ▶). 

The wells were made on agar plates with 5 mm diameter. 125, 250, 500 and 1000 mg of the methanol extract and the fractions were separately dissolved in 1 ml DMSO (10%) and then filtered and 80 μl of each solution was added to each well. Ciprofloxacin (40 mg/ml) and Gentamycin (40 mg/ml) were used as positive controls for Gram-positive and Gram-negative microorganisms respectively. Eighty μl of pure DMSO (10%) served as negative control. The plates incubated at 37º C for 24 h .The diameter of zone of inhibitions was detected in each plate. The experiments carried out three times and the results were presented as mean±SD.

**Minimum inhibitory concentration (MIC)**

After confirmation of the antibacterial activity in the extract and the obtained fractions, MIC of each was determine using the ten testing concentrations of the extract and every fraction against sensitive Gram-positive and Gram-negative tested bacteria by the micro plate dilution method. The reconstituted extract was diluted to give concentrations of 0.48, 0.97, 1.95, 3.90, 7.81, 15.62, 31.25, 62.5, 125 and 250 mg/ml. The lowest concentration of the extract that could inhibit the bacterial growth was considered as MIC (Mehregan et al., 2008 ▶). Similarly, Erythromycin and Gentamycin, and pure DMSO (10%) were used as positive and negative controls, respectively.

**Antifungal assay**

The total extract and alkaloid sub-fraction were prepared by dissolving in their specific solvents (methanol and chloroform), after which, they were loaded into blank paper disks at the concentrations of 8, 4, 2, 1 and 0.5 mg/disc. Nystatin (10 μg/disc) was used as positive control.

The isolates were transferred from DW (distilled water) stocks to Mycosel agar and then sub-cultured to Potato dextrose agar (Merck, Germany) to enhance sporulation. Seven day-old cultures were covered with 1 ml DW and the colonies were probed with the tip of a sterile Pasteur pipette to obtain a mixture of mycelium and conidia. The suspensions were transferred to sterile tubes and allowed to sediment for 30 min and then adjusted with a spectrophotometer set at 65% transmittance and 530 nm (Esteban et al., 2005 ▶).

All tests were performed according to Esteban et al. (Esteban et al., 2005 ▶). The inoculum was evenly spread on the surface of 10 cm Petri dishes containing Sabouraud dextrose agar medium (Merck, Germany) and exposed to air dry. Then, the antifungal disks were applied to the plates, after which the plates were incubated at 25° C for 5-10 days. After the colonies grew, the zones of inhibition around the disks were measured and recorded. Criteria of susceptibility and resistance of antifungal disks were measured according to Table 3. All tests were performed in triplicate and Microsoft SPSS was used for data analysis (Pakshir et al., 2009 ▶).

## Results

According to Table 1, *G. vitellinum* crude extract and alkaloid sub-fraction had significant inhibition activity on the growth of *S. aureus* and *S. typhi* while weak antibacterial activity was observed against *E. coli, K. pneumonia *and* P. aeroginosae *and sub-fraction were determined for the most sensitive strains (Table 2). 

The results of antifungal assay revealed that the investigated alkaloid sub-fraction was active just against *C. albicans* and it was concluded that the plant was totally inactive against *T. mentagrophytes, T. rubrum, Aspergillus flavus, Epidermophyton flucossum *and* Microsporum canis* (Table 3). MIC values of the extract of the alkaloid sub-fraction against *C. albicans* were determined as 10 mg/ml and 0.02 mg/ml respectively. MIC value for Nystatine was equal to 0.02 mg/ml.

**Table 1:** The inhibition zone diameter of total extract and alkaloid sub-fraction of *G. vitellinum*

	**Diameter of zone of inhibition (mm)**									
	**MTE** (mg/ml)				**ASF **(mg/ml)				**Gen.**	**Cipr**.
*S. aureus*	125	250	500	1000	125	250	500	1000	40	40
*E. coli*	10.6	10.3	11.3	10.6	15.4	17.6	18.3	19.0	-	28.1
*S. typhi*	-	-	-	7.1	6.5	7.0	7.3	9.3	25.5	-
*P. aeruginosa*	11.6	12.0	13.7	14.1	13.9	15.2	17.3	17.8	19.5	-
*S. aureus*	-	-	-	-	6.7	7.0	7.7	8.0	16.5	-

MTE=Methanol total extract; ASF=Alkaloid sub-fraction.Gen.=Gentamycin; Cipr.=Ciprofloxacin

ª Zone of inhibition, including the diameter of the well (6 mm); mean value of three independent experiments.

**Table 2:** Minimum inhibitory concentration (MIC) of total extract and alkaloid sub-fraction of *G. vitellinum*ª

	**MIC (mg/ml)**			
	**MTE**	**ASF**	**Gen.**	**Cipr**
*S. aureus *	>1000	0.9	-	0.4
*S. typhi *	1000	11.7	0.4	-

MTE=Methanol total extract; ASF=Alkaloid sub-fraction.

ª All determinations were done in triplicate.

**Table 3:** The inhibition zone diameter of total extract and alkaloid sub-fraction of *G. vitellinum*

	**Diameter of zone of inhibition (mm)**									
	**MTE** (mg/ml)				**ASF **(mg/ml)				**Gen.**	**Cipr**.
*S. aureus*	125	250	500	1000	125	250	500	1000	40	40
*E. coli*	10.6	10.3	11.3	10.6	15.4	17.6	18.3	19.0	-	28.1
*S. typhi*	-	-	-	7.1	6.5	7.0	7.3	9.3	25.5	-
*P. aeruginosa*	11.6	12.0	13.7	14.1	13.9	15.2	17.3	17.8	19.5	-
*S. aureus*	-	-	-	-	6.7	7.0	7.7	8.0	16.5	-

MTE=Methanol total extract; ASF=Alkaloid sub-fraction. Nys.=Nystatin.

ª Zone of inhibition, including the diameter of the well (6 mm); mean value of three independent experiments.

## Discussion

Occurrence of bacterial and fungal diseases is a serious problem in the present world. This is because of the development of drug resistance of the pathogens and side effects exhibited by the drugs used for curing bacterial and fungal diseases.

Hence there is a great demand for safer, alternative and effective therapeutic agents. Application of medicinal plants to treat bacterial and fungal infections is an old practice in many parts of the world (Irobi and Darambolo, 1993 ▶). 

Plants contain a spectrum of secondary metabolites that their importance as antimicrobial or antifungal agents has been emphasized by several investigations (Vaijayantimala et al., 2001 ▶).

As the endemic species *G. vitellinum *is used traditionally as a disinfectant and antifungal agent in dermatitis, we prompted to evaluate the antibacterial and antifungal activities of the plant against eleven bacterial and fungal strains. Total methanol extract and the alkaloid sub-fraction had inhibitive activities on the growth of *S. aureus* and *S. typhi* while weak antibacterial activity was observed against remaining studied strains. The inhibition zone diameters of the active ones were measures in the range of 19-10 mm compared with the positive standards, Gentamicine and Ciprofloxacin (28-16 mm).

According to Table 3 the crude extract and the alkaloid sub-fraction were both completely inactive against the investigated fungal strains except for the yeast *C. albicans. *It was observed that *C. albicans* showed sensitivity to both of the extract and the sub-fraction by inhibition zone diameters 18.3-3.7 mm. The strongest antifungal activity against *C. albicans* was observed by the alkaloid fraction. 

Phytobiological evaluations of *Glaucium* species showed that the alkaloid content of these plants had marked antibacterial and antifungal properties (Morteza-Semnani et al., 2005 ▶; Coşar et al., 1981 ▶). Literature survey revealed that other *Glaucium* species, *G. grandiflorum G. oxylobum*, and *G. paucilobum* showed good antibacterial activity against Gram positive and negative bacterial strains (Moterza-Semnani et al., 2005 ▶). The most significant effect was observed by the chloroform sub-fractions which were rich in alkaloid. 

As it is observed from Table 3, the alkaloid sub-fraction was effective only against *C. albicans* with an efficacy near to the antifungal drug, Nystatin. It was expectable because it was found in the previous studies the alkaloids of other *Glaucium* species shown good antifungal activities. 

Results of the current study showed that *G. vitellinum* possess reliable antibacterial and antifungal activities related to its alkaloid content. This plant could be a good choice for more complete in vitro studies and then further in vivo biological investigations especially on the antifungal properties of the alkaloids present in the plant. 
